# QuickStats

**Published:** 2013-05-24

**Authors:** Cheryl D. Fryar

**Figure:**
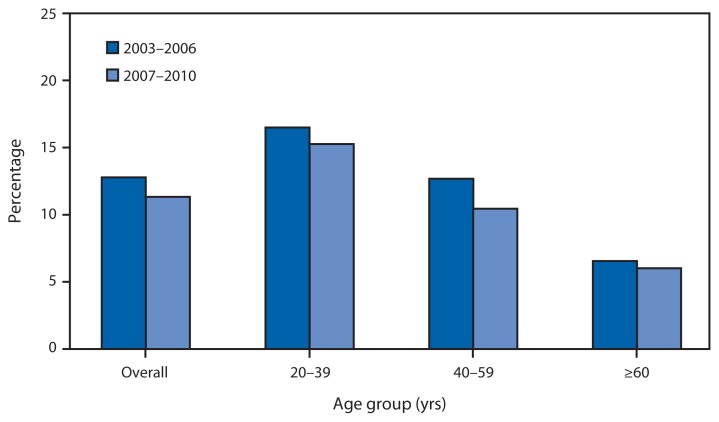
Percentage of Daily Calories Consumed from Fast Food^*^ Among Adults Aged ≥20 Years, by Age Group^†^ — National Health and Nutrition Examination Survey, United States, 2003–2006 and 2007–2010 ^*^ Food usually sold at eating establishments for quick availability or takeout. ^†^ Overall estimates age adjusted to year 2000 U.S. Census standard population using age groups 20–39 years, 40–59 years, and ≥60 years.

From 2003–2006 to 2007–2010 the percentage of daily calories consumed from fast foods among adults aged ≥20 years declined from 12.8% to 11.3%. A decrease from 12.7% to 10.5% also was observed for those aged 40–59 years, but no statistically significant change was noted for persons aged 20–39 years or ≥60 years. During both periods, the percentage of daily calories from consumption of fast foods was highest among those aged 20–39 years.

**Source:** Fryar CD, Ervin RB. Caloric intake from fast food among adults: United States, 2007–2010. NCHS data brief no. 114. Hyattsville, MD: US Department of Health and Human Services, CDC; 2013. Available at http://www.cdc.gov/nchs/data/databriefs/db114.pdf.

